# Substance use and adolescent mental health during the COVID-19 pandemic in Brazil: a longitudinal approach

**DOI:** 10.1016/j.jped.2024.01.005

**Published:** 2024-04-03

**Authors:** Rodrigo Garcia-Cerde, Gabriela A. Wagner, Juliana Y. Valente, Zila M. Sanchez

**Affiliations:** Universidade Federal de São Paulo, Departamento de Medicina Preventiva, São Paulo, SP, Brazil

**Keywords:** Latin America, Psychiatric symptomatology, Longitudinal study, Substance use

## Abstract

**Objective:**

To describe the changes in alcohol and drug use by Brazilian adolescents during the COVID-19 pandemic (April-August 2021), and to analyze the relationship between alcohol use changes and psychiatric symptomatology.

**Methods:**

A secondary analysis with a longitudinal approach was performed with data from a cluster-randomized controlled trial, conducted in 73 public middle schools in three Brazilian cities, to evaluate the effectiveness of a drug use prevention program. The sample included 535 students (61% girls; *M*_age_ = 15.2 years). Data were collected pre-intervention (February-March 2019), after 9 months (November-December 2019), and after 26 months (April-August 2021), when the students were in their first year of high school. The authors analyzed drug use prevalence (alcohol, binge drinking, tobacco, inhalants, marijuana, cocaine, and crack) in a lifetime, past year, and past month periods, and the association between alcohol use change subsamples with psychiatric symptoms. Logistic regressions were adjusted by sex, age, socioeconomic status, city, and group (control and intervention).

**Results:**

The present findings suggest that the COVID-19 pandemic led to a decrease in past-year substance use and in past-month substance use frequency, despite the gradually increased (but decelerating) prevalence of lifetime use. However, some adolescents initiated, maintained, or increased the frequency of their alcohol use. Mainly, they were more likely to present behavioral problems, as well as symptoms of inattentive hyperactivity, and peer and emotional problems.

**Conclusions:**

Despite the extensive decline in substance use during the pandemic period, these results suggest an association between previous mental health conditions and behavioral risk factors, leading to increased alcohol consumption and behavioral disorders manifestations.

## Introduction

Studies on drug use in adolescents before, during, and after the most restrictive period of the COVID-19 pandemic have shown mixed results.^1^ Some studies have reported increased prevalence and frequency of drug use in adolescents or subpopulations, and others showed a general decrease or no substantial change among drug users. For example, in Canada,^2^^,^^3^ the United States,^4^^,^^5^ and Europe^6^^,^^7^ an increase and decrease in drug use frequency in adolescents was reported before, during, and shortly after the most restrictive period of the COVID-19 pandemic. Studies in Australia^8^ and Israel^9^ described an increase in frequency. In Uganda,^10^ an overall decrease was observed, characterized by a slowdown in the expected increase in drug use among this population.

This great heterogeneity in the literature on the subject can be explained by methodological differences and deficiencies according to public health and adopted health measures during the pandemic, socioeconomic disparity, political and financial instability, demographic density, previous epidemiological profile of substance use, and mental health conditions.^11^ Despite the variety of results, most studies found a generalized decrease in drug use among adolescents, suggesting an increased frequency of drug use among adolescents with prior risk factors or with vulnerable conditions developed during the COVID-19 pandemic. On this point, Temple et al.^12^ noted that teens who did not limit their social gatherings during the pandemic were more likely to use drugs.

The literature on the effects of the pandemic on adolescent mental health is extensive and suggests the onset or exacerbation of emotional, psychological, and psychiatric symptoms during this period, the effects of which could be prolonged in the long term.^13^ Recent evidence shows more frequent pandemic effects among young people, such as anxiety, depression, sleep disturbances, loneliness, post-traumatic stress disorders, fear, tension, anger, fatigue, confusion, and worry.^14^ Other studies in different countries have also found increases in symptoms of depression, anxiety, and stress, suicide attempts,^15^ and social problems.^2^^,^^6^^,^^12^^,^^16^^,^^17^ Few studies have reported no changes.^3^

Regarding the relationship between the negative effects of the COVID-19 pandemic on adolescent mental health and substance use, associations have been observed between anxiety and problematic alcohol use,^5^ depression and substance-related coping,^18^ and severe stress, depression, or anxiety with substance use.^19^ Despite there being a significant amount of literature on the subject, as far as the authors know, no published studies have addressed the changes produced by the pandemic simultaneously in drug use and mental health, and their association, among Latin American adolescents.

To contribute to the understanding of the changes produced by the pandemic period in the drug use of Latin American adolescents and its relationship with their mental health, the authors defined two objectives for this study. The first is to describe the changes in the use of alcohol and other drugs by Brazilian adolescents related to the COVID-19 pandemic period. The second was to analyze the association between changes in alcohol use and psychiatric symptomatology in the follow-up during the pandemic.

## Methods

### Study design and sampling

For this study the authors used data from a cluster-randomized controlled trial (cRCT) conducted to evaluate the effectiveness of the #*Tamojunto2.0* program, a school-based intervention for drug use prevention in the adolescent population, with eighth-grade students in 73 middle public schools in the Brazilian cities of São Paulo, Fortaleza, and Eusébio. Details on the study design, randomization, and sampling methods can be found in a previous publication.^20^ Originally, the study was designed to have 3 in-person data collections in the classrooms of the selected schools. However, due to restrictions imposed by the COVID-19 pandemic, the third collection had to be reconsidered and was finally conducted with an online survey. The sample number obtained was 535 valid questionnaires. The original purpose was to evaluate the effects of the program at 9 (T2) and 18 months (T3). However, during the pandemic, the 18-month follow-up was suspended and carried at 26 months. In the end, the T3 data collection did not generate enough data to allow the investigation of the effects of the program, since the sample loss was about 90%. However, the authors could analyze predictors of outcomes during the pandemic, by using data from the baseline, T2, and T3 and controlling results per randomization group, using the T3 participants' data. In this way, the authors used information from the 535 participants for whom we have data at all three collection times. At the baseline, the students were in the 8th grade, and during T3 they were in the first year of high school.

### Data collection

The baseline was conducted before implementing the program in February and March 2019 (T1), the first follow-up data was collected nine months after the baseline in November and December 2019 (T2), and the second follow-up was scheduled to take place in August 2020. However, during the pandemic, the suspension of in-person classes made it impossible to carry out the T3 data collection, since schools remained closed. As mentioned above, in order not to lose the opportunity to collect the third wave, the authors opted for a remote collection between April and August 2021 (T3).

### Ethical considerations

Written informed consent to participate in the study was obtained from the school directors before randomization and from students and parents after randomization. This trial and the pre-registered hypothesis were registered in the Brazilian Registry of Clinical Trials (RBR-8cnkwq). The protocol was approved by the Universidade Federal de São Paulo Research Ethics Committee (#2,806,301) and the Ethics Committee of the Municipal Health Secretariat (#3,099,865).

### Measurements

In the baseline and in the 9-month follow-up, an anonymous paper-and-pencil questionnaire was completed by the students and administered by researchers without a teacher in the classroom. For the 26-month follow-up, a questionnaire software was created, and each school received an individual collection link (one link per school). In each assessment, students provided a code generated from letters and numbers from their personal information. The datasets of the three evaluation time points were integrated by matching this confidential code.

The instrument was the same as the one used in the previous evaluation studies of #*Tamojunto* and *Unplugged*. The Brazilian Portuguese version was adapted, supplemented, and validated.^21^ To avoid over-reporting of drug use, the authors excluded questionnaires that were positive for lifetime use of a fictional drug (Holoten and Carpinol) from the analysis (baseline, n = 35; follow-up, n = 37).

### Outcomes

The drug use variables were lifetime, past year, and past month use of alcohol, binge drinking (the consumption of five or more doses of alcohol in two hours), tobacco, inhalants, marijuana, cocaine, and crack. Questions for lifetime and past year use were formulated dichotomously (*Yes/No*). For example, “Have you ever tried an alcoholic drink?” or “In the last 12 months, did you drink any alcoholic beverages?” Questions for past month use were formulated to inquire about recent consumption frequency, as follows: “In the last 30 days, did you drink any alcoholic beverages?” and were answered polytomously (*No/ 1 to 5 days/ 6 to 19 days/ 20 or more days*).

The dichotomous variables of change in drug use were constructed arithmetically by using the corresponding drug use variables from T1 and T3. The category coded with the value “1″ represents individuals who changed or maintained their use at T3, whereas the category coded with the value “0″ represents individuals who were abstainers at both T1 and T3. Thus, for the “lifetime use” variables, the authors obtained the variable representing the subsample of those who initiated use at T3. For the “past year use” variables, the authors obtained the subsamples of those who started, stopped, and maintained their use. For the “past month use” variables, the authors obtained the subsamples of those who increased, decreased, and maintained their use frequency.

### Covariates

The Strengths and Difficulties Questionnaire (SDQ)^22^ was used to assess the probable psychiatric symptoms of the participants. The SDQ is a screening measure of emotional/behavioral difficulties (20 items) and prosocial skills (5 items) for 4- to 17-year-olds. It comprises five subscales: emotional symptoms, conduct problems, inattention-hyperactivity symptoms, peer problems, and prosocial behavior. Each subscale has five items that can be answered with “not true,” “somewhat true,” or “certainly true.” The prosocial behavior subscale was excluded from the total score since it does not assess symptoms or problems, but rather prosocial resources or skills. This questionnaire has already been translated into Portuguese and has been validated for Brazilian children and adolescents in terms of discriminative validity, reliability, and internal consistency. The participants were classified according to their total score as “non-case” (normal), “subclinical” (borderline), and “case” (abnormal). Adolescents were also classified into these categories on each subscale. The “non-case” category was used as the reference in all models.

The sociodemographic characteristics were sex (boys/girls), age, socioeconomic status, city, and group (control/intervention). Students’ socioeconomic status was assessed with the scale of the Brazilian Association of Research Companies (ABEP), which is scored from 1 to 100 points and considers the education level of the head of the household and the goods and services used, with categories ranging from A (highest) to D/E (lowest).

### Statistical analysis

First, the sociodemographic characteristics and prevalence of the SDQ scale and subscales at baseline, 9-month, and 26-month follow-ups were described by using proportions or means. Univariate analyses between each collection time were performed by using the *t*-test for paired samples and the chi-squared test, presenting the p-value with a level of significance set at 5%. This was followed by an exploratory and descriptive analysis comparing the prevalence of substance use in each collection time and the change in drug use subsamples obtained arithmetically, which were described using proportions. Finally, separate logistic regression models were performed to estimate the association between change in alcohol use subsamples and SDQ scale and subscales, controlling by sex, age, socioeconomic status, city, and group. The “non-case” category of the SDQ scale/subscales was the reference in all models. The authors decided to model only the variables of change in alcohol use since they had the largest sample size compared with other drugs. Results are presented as adjusted odds ratios (aOR), 95% confidence intervals (95%CI), and p-values with a 5% level of significance. All statistical analyses were conducted using Stata version 7 (StataCorp LP, College Station, TX, USA).

## Results

Table 1 shows the sociodemographic characteristics and prevalence of SDQ scale and subscales at baseline and both follow-ups. Most of the sample was female (61%), with an average age of 15.2 years at the second follow-up, mainly from the city of Eusébio (39%), and over half of it belonged to the intermediate socioeconomic class (53%) and from the intervention group (54%). Compared with the baseline, at 26-month follow-up the authors observed an increase in the prevalence of SDQ scale for the “cases” category (+11%) as well as for the emotional symptoms (+10%), inattention-hyperactivity symptoms (+10%), and peer problems (+4%) subscales. Only the behavioral problems subscale showed a 7% decrease in the prevalence of cases.

**Table 1:** Sociodemographic characteristics and prevalence of SDQ scale and subscales at baseline, 9-month, and 26-month follow-up (*n* = 535).

Variables	Baseline (T1) 8th grade Middle school	9 months (T2) 8th grade Middle school	26 months (T3) 1st grade High school	
	n/N (%)	n/N (%)	n/N (%)	p-value 1
Sex				
Boys	207/529 (39.13)	–	–	
Girls	322/529 (60.87)	–	–	
Age (±SD)	12.98±0.73	13.59±0.74	15.20±0.86	
Socioeconomic Status				
Average SES score (±SD)	23.76±9.01	–	–	
A: 45–100 (highest)	14/532 (2.63)	–	–	
B: 29 – 44	122/532 (22.93)	–	–	
C: 17 – 28	283/532 (53.20)	–	–	
D/E: 1 – 16 (lowest)	113/532 (21.24)	–	–	
City				
Eusébio	211/535 (39.44)	–	–	
Fortaleza	197/535 (36.82)	–	–	
São Paulo	127/535 (23.74)	–	–	
Group				
Control	248/535 (46.36)	–	–	
Intervention	287/535 (53.64)	–	–	
				
SDQ total				
Non-case	254/441 (57.60)	251/452 (55.53)	200/421 (47.51)	<0.001 2
Subclinical	94/441 (21.32)	81/452 (17.92)	88/421 (20.90)	
Case	93/441 (21.09)	120/452 (26.55)	133/421 (31.59)	
SDQ subscales				
Emotional symptoms				
Non-case	300/444 (67.57)	287/454 (63.22)	243/421 (57.72)	<0.001 2
Subclinical	56/444 (12.61)	51/454 (11.23)	52/421 (12.35)	
Case	88/444 (19.82)	116/454 (25.55)	126/421 (29.93)	
Conduct problems				
Non-case	289/444 (65.09)	298/454 (65.64)	306/421 (72.68)	0.034 2
Subclinical	64/444 (14.41)	69/454 (15.20)	60/421 (14.25)	
Case	91/444 (20.50)	87/454 (19.16)	55/421 (13.06)	
Inattention-hyperactivity symptoms				
Non-case	308/441 (69.84)	304/453 (67.11)	249/421 (59.14)	<0.001 2
Subclinical	58/441 (13.15)	66/453 (14.57)	59/421 (14.01)	
Case	75/441 (17.01)	83/453 (18.32)	113/421 (26.84)	
Peer problems				
Non-case	252/443 (56.88)	272/453 (60.04)	206/421 (48.93)	0.040 2
Subclinical	132/443 (29.80)	121/453 (26.71)	143/421 (33.97)	
Case	59/443 (13.32)	60/453 (13.25)	72/421 (17.10)	

1 All hypothesis tests were performed by comparing the baseline measurement with that of time 3.

2 McNemar's Chi-Square Test.

Table 2 shows the prevalence of drug use and changes in drug use at lifetime, past year, and past month at baseline (T1), 9-months (T2), and 26-month follow-up (T3). **Lifetime drug use** variables showed changes from T1 to T3 in all substances. However, a greater difference can be observed between T1 and T2 prevalence, compared with those of T2 and T3. This could indicate a slowdown in the gradual increase observed in substance use prevalence among adolescents. The highest prevalence of drug use onset was observed for alcohol, binge drinking, and inhalants. Regarding **past year use**, a trend of increase at T2 and decrease at T3 was observed for all drugs. Despite this consistent trend, individuals who initiated and maintained their use were also observed, but people who stopped their consumption had the highest proportions.

**Table 2:** Prevalence of drug use and changes in drug use in lifetime, past year, and past month at baseline, 9-months, and 26-months follow-up (*n* = 529).

Period of use	Samples	Alcohol		Binge drinking		Tobacco		Inhalants		Marijuana		Cocaine		Crack	
		Freq.	%	Freq.	%	Freq.	%	Freq.	%	Freq.	%	Freq.	%	Freq.	%
Lifetime	Baseline (T1)	213/531	40.11	74/533	13.88	48/532	9.02	98/532	18.42	21/530	3.96	3/529	0.57	0/528	0
	9 months (T2)	256/466	54.94	117/465	25.16	80/465	17.20	130/464	28.02	38/466	8.15	6/461	1.30	2/461	0.43
	26 months (T3)	337/531	63.47	159/514	30.93	110/503	21.87	164/509	32.22	54/499	10.82	9/490	1.84	2/493	0.41
	Started using at T3^1^	121/527	22.96	85/512	16.60	62/500	12.40	66/506	13.04	33/494	6.68	6/484	1.24	2/487	0.41
Past year	Baseline (T1)	134/534	25.09	61/534	11.42	26/531	4.90	49/532	9.21	16/529	3.02	1/529	0.19	–	–
	9 months (T2)	130/466	27.90	77/464	16.59	33/466	7.08	61/465	13.12	24/465	5.16	2/461	0.43	–	–
	26 months (T3)	122/513	23.78	36/502	7.17	24/499	4.81	12/496	2.42	15/496	3.02	0/490	0	–	–
	Started using at T3^1^	70/512	13.67	26/501	5.19	17/495	3.43	5/493	1.01	10/490	2.04	0/484	0	–	–
	Stopped using at T3^2^	74/512	14.45	45/501	8.98	19/495	3.84	38/493	7.71	11/490	2.24	1/484	0.21	–	–
	Maintained using at T3^3^	52/512	10.16	10/501	2.00	6/495	1.21	7/493	1.42	5/490	1.02	0/484	0	–	–
Past month	Baseline (T1)	76/534	14.23	49/534	9.18	10/532	1.88	25/529	4.73	10/529	1.89	0/530	0	–	–
	9 months (T2)	72/466	15.45	58/464	12.50	22/466	4.72	26/466	5.58	16/465	3.44	1/462	0.22	–	–
	26 months (T3)	56/510	10.98	21/479	4.38	8/473	1.69	2/450	0.44	6/482	1.24	0/487	0	–	–
	Increased use frequency at T3^4^	38/509	7.47	12/473	2.54	6/470	1.28	1/444	0.23	3/476	0.63	0/482	0	–	–
	Decreased use frequency at T3^5^	59/509	11.59	37/473	7.82	5/470	1.06	16/444	3.60	5/476	1.05	0/482	0	–	–
	Maintained same use frequency at T3^6^	11/509	2.16	1/473	0.21	2/470	0.43	1/444	0.23	3/476	0.63	0/482	0	–	–

1 This is a subsample composed by abstainers at time 1 who started using at time 3.

2 This is a subsample composed by users at time 1 who stopped using at time 3.

3 This is a subsample composed by users at time 1 who maintained their use at time 3.

4 This is a subsample composed by users at time 1 who increased their frequency of use at time 3 and abstainers at time 1 who started using at time 3.

5 This is a subsample composed by users at time 1 who decreased their frequency of use at time 3 and users at time 1 who stopped their use at time 3.

6 This is a subsample composed by users at time 1 who maintained the same frequency of use at time 3. Abbreviations: “Freq”: absolute frequency.

**The past month's drug use** variables showed the same results. At T2, an increase was observed, but at T3 it was reversed. This suggests that COVID-19 pandemic isolation measures led to a decrease in substance use prevalence at short-term, and a decrease in recent substance use frequency. Despite this trend, some participants increased and maintained their frequency of use, although the highest proportion was observed among those whose use decreased. The highest prevalence was always for alcohol consumption in all type of frequency use (Table 2).

Table 3 shows the outcomes of logistic regression models evaluating the association between alcohol use changes at T3 and SDQ scale and subscales. Compared to abstainers, those who initiated their alcohol consumption, lifetime and past year, were more likely to be characterized as “cases” on the general SDQ scale and to have some type of behavioral problem or inattentive-hyperactivity symptoms. Additionally, those who stopped using alcohol in the past year, had more probability of being subclinical in the SDQ scale; and those who maintained using alcohol in the past year, were more likely to be “case” and “subclinical” in behavioral problem and peer problem subscales, respectively. Regarding past month alcohol use variables, those who increased their use frequency were “cases” in emotional and conduct problems subscales; those who decreased the alcohol use had no type of peer problem symptoms; and those who maintained their use were more likely to be “cases” in the behavioral problem subscale.

**Table 3:** Outcomes of logistic regression models evaluating the association between alcohol use changes at 26-months follow-up and SDQ scale and subscales.

	Lifetime use^1^			Past year use^1^								
SDQ categories	Started using alcohol *versus* Abstainers^2^			Started using alcohol *versus* Abstainers^2^			Stopped using alcohol *versus* Abstainers^2^			Maintained using alcohol *versus* Abstainers^2^		
	aOR	95%CI	p-value	aOR	95%CI	p-value	aOR	95%CI	p-value	aOR	95%CI	p-value
												
SDQ total												
Non-case	1			1			1			1		
Subclinical	1.26	[0.60; 2.66]	0.546	0.95	[0.38; 2.41]	0.921	2.15	[1.04; 4.44]	**0.038**	1.84	[0.71; 4.78]	0.209
Case	2.44	[1.31; 4.55]	**0.005**	2.58	[1.32; 5.04]	**0.006**	0.97	[0.46; 2.05]	0.929	2.21	[0.97; 5.03]	0.058
												
Emotional symptoms												
Non-case	1			1			1			1		
Subclinical	0.96	[0.42; 2.19]	0.914	1.16	[0.45; 2.97]	0.754	0.86	[0.34; 2.15]	0.741	1.04	[0.34; 3.17]	0.942
Case	1.47	[0.78; 2.76]	0.233	1.71	[0.87; 3.37]	0.121	1.04	[0.52; 2.08]	0.919	1.88	[0.86; 4.11]	0.113
												
Conduct problems												
Non-case	1			1			1			1		
Subclinical	2.69	[1.18; 6.14]	**0.019**	2.88	[1.30; 6.38]	**0.009**	1.74	[0.77; 3.93]	0.186	1.88	[0.62; 5.67]	0.264
Case	3.31	[1.35; 8.12]	**0.009**	4.33	[1.90; 9.88]	**<0.001**	1.41	[0.51; 3.88]	0.507	7.72	[3.23; 18.49]	**<0.001**
												
Inattention-hyperactivity symptoms												
Non-case	1			1			1			1		
Subclinical	2.47	[1.12; 5.44]	**0.025**	2.11	[0.93; 4.77]	0.072	0.91	[0.36; 2.33]	0.851	0.61	[0.21; 1.90]	0.371
Case	2.20	[1.17; 4.13]	**0.014**	2.11	[1.06; 4.20]	**0.033**	1.58	[0.82; 3.06]	0.175	0.87	[0.38; 1.97]	0.737
												
Peer problems												
Non-case	1			1			1			1		
Subclinical	1.29	[0.70; 2.39]	0.411	1.96	[0.99; 3.85]	0.052	1.27	[0.65; 2.46]	0.482	2.74	[1.22; 6.11]	**0.014**
Case	1.51	[0.71; 3.23]	0.286	2.05	[0.91; 4.60]	0.082	1.12	[0.48; 2.60]	0.797	2.48	[0.95; 6.44]	0.063

	Past month use^1^											
SDQ categories	Increase using alcohol *versus* Abstainers^2^			Decrease using alcohol *versus* Abstainers^2^			Maintained using alcohol *versus* Abstainers^2^					
	aOR	95%CI	p-value	aOR	95%CI	p-value	aOR	95%CI	p-value			
SDQ total												
Non-case	1			1			1					
Subclinical	1.50	[0.48; 4.75]	0.487	1.20	[0.54; 2.68]	0.658	1.77	[0.23; 13.53]	0.583			
Case	2.39	[0.93; 6.12]	0.070	1.09	[0.53; 2.25]	0.815	2.26	[0.39; 13.14]	0.362			
Emotional symptoms												
Non-case	1			1			1					
Subclinical	1.18	[0.30; 4.61]	0.814	0.88	[0.33; 2.32]	0.793	–	–	–			
Case	2.83	[1.14; 7.03]	**0.026**	1.19	[0.59; 2.42]	0.631	2.13	[0.46; 9.93]	0.337			
Conduct problems												
Non-case	1			1			1					
Subclinical	2.49	[0.86; 7.25]	0.093	0.60	[0.20; 1.79]	0.362	1.75	[0.17; 18.24]	0.640			
Case	5.71	[2.20; 14.78]	**<0.001**	1.59	[0.66; 3.80]	0.300	11.63	[2.26; 59.80]	**0.003**			
Inattention-hyperactivity symptoms												
Non-case	1			1			1					
Subclinical	0.88	[0.26; 3.01[	0.845	0.72	[0.27; 1.87]	0.493	–	–	–			
Case	1.72	[0.71; 4.14]	0.226	1.03	[0.50; 2.10]	0.943	1.26	[0.28; 5.62]	0.761			
Peer problems												
Non-case	1			1			1					
Subclinical	1.56	[0.64; 3.81]	0.333	2.21	[1.07; 4.57]	**0.032**	1.21	[0.26; 5.69]	0.807			
Case	1.46	[0.49; 4.36]	0.494	2.55	[1.11; 5.83[	**0.027**	0.60	[0.06; 5.93]	0.666			

1 All models were adjusted by sex, age, socioeconomic status, city, and group (control and intervention).

2 This reference category is composed by abstainers at time 1 who continued to be abstinent at time 3. Abbreviations: “aOR”: Adjusted Odds Ratio; “95%CI”: 95% Confidence Interval; “SES”: Socioeconomic Status; “SDQ”: Strengths and Difficulties Questionnaire.

## Discussion

This study aims to describe the changes in drug use by Brazilian adolescents related to the COVID-19 pandemic and to investigate the relationship between alcohol use changes and psychiatric symptomatology at this period. The present findings suggest that COVID-19 pandemic isolation measures led to a decrease (from T2 to T3) in the past year's substance use, and in the past month's substance use frequency, despite the gradually increased but decelerated lifetime drug use (expected increase from T1 to T3). On the other hand, those who initiated or maintained their use, or increased their frequency use of alcohol during the pandemic period were more likely to have behavioral problems and to present some symptoms of inattentive-hyperactivity, peer problems, or emotional problems.

The descriptive results suggest a slowdown in the experimentation of new substances. These results are consistent with previous evidence regarding the declining prevalence of drug use during the COVID-19 pandemic for alcohol and tobacco.^1^^,^^3^^,^^4^^,^^6^ One hypothesis for these results may be that this reduction is due to greater parental support/supervision and decreased availability of substances attributed to containment measures.^4^^,^^19^

The findings also corroborate the increase in the prevalence and frequency of substance use among adolescents with certain risk factors or vulnerabilities, such as the occurrence of mental disorders. For example, an online survey conducted in Canada among adolescents with poorer mental health and coping showed a lower proportion of drug users but reported an increase in the frequency of alcohol and marijuana use.^23^

These results on the onset, maintenance, or increase in the frequency of alcohol use (despite the general tendency of decreasing it throughout life), may reflect an association of mental and behavioral risk factors prior to the pandemic period,^24^ leading to increased alcohol consumption with manifestations of behavioral changes.^1^ Alcohol use among adolescents who did not limit their social interaction during the pandemic was evaluated in the United States. This study showed an increase in the use of many substances, with a greater propensity for alcohol use among those who worsened their financial situation.^12^ In addition, other longitudinal studies in the same country have shown that more than 80% of adolescents who used substances during the pandemic were already users, making the use more harmful to these individuals.^25^

In this sense, the present findings characterized students who started or continued using alcohol or who increased their frequency of consumption. The authors observed that most of them presented mainly symptoms of behavioral problems, but also inattentive-hyperactivity problems, problems with colleagues, or emotional problems. Behavioral problems and their association with drug use during the pandemic may be related to previous problems at home and the lack of adequate coping strategies to deal with the social contact restrictions imposed by COVID-19.^26^ In this context, these findings corroborate previous research in Australia and Spain, which found that about 26% of children and adolescents presented behavioral problems in the early phase of COVID-19^27^ and significantly worse emotional symptoms, behavioral problems, hyperactivity/inattention, and problems with peers in adolescents.^28^ However, they differ from the evidence found in Norway, where behavioral problems remained stable for a sample of adolescents representative of the population.^7^

Particularly, regarding the relationship between substance use and problems with peers, the authors hypothesized it as an attempt to improve social integration with peers during social distancing, as already demonstrated in Canada, where adolescents with low self-reported popularity were more likely to use drugs in person, and those with medium and high popularity used drugs alone.^23^ Other studies have also shown that children and adolescents living with internalizing and externalizing symptoms — such as anxiety or depression and inattention-hyperactivity or relationship problems — were especially vulnerable to negative effects on mental health and to single-use and polydrugs during the COVID-19 pandemic.^26^^,^^29^

The present study is the first to investigate the Latin American adolescent population to analyze the consequences of drug use and its relationship with mental health. Moreover, this is a study with a longitudinal approach in which adolescents were matched before and during the COVID-19 pandemic. However, it has limitations, which should be considered: (a) due to social distancing measures in the first phase of the COVID-19 pandemic, the third wave of data collection was not conducted in person like the two previous collections, pre-pandemic; (b) accessing the students was very difficult, which limited the sample and the ability to extrapolate these results; (c) since this is a descriptive-observational study, establishing causality between the presence of psychiatric symptoms and the use of substances during the pandemic period is not possible; in particular because the analysis strategy the authors used is not strictly longitudinal, although we did implement a robust methodology with measures of change over time.

The present results may assist in executing preventive actions regarding the long-term negative consequences among children and adolescents since they are more vulnerable than adults to the development of physical, psychological, behavioral, and social conditions related to the COVID-19 pandemic.^30^ One strategy would be to train public elementary and high school teachers so that they can identify students’ mental health needs and refer them to appropriate health services. These efforts are urgently needed considering the possibility that the negative health effects of the pandemic could be far-reaching among adolescents and young people, many of whom have transitioned from primary education to secondary education during the pandemic, which can be a more stressful time of emotional and social transition.

## Conflicts of interest

The authors declare no conflicts of interest or personal relationships (real and perceived) that could have appeared to influence the work reported in this paper.
